# Preoperative tumor size measurement in breast cancer patients: which threshold is appropriate on computer-aided detection for breast MRI?

**DOI:** 10.1186/s40644-020-00307-0

**Published:** 2020-04-28

**Authors:** Sung Eun Song, Bo Kyoung Seo, Kyu Ran Cho, Ok Hee Woo, Eun Kyung Park, Jaehyung Cha, Seungju Han

**Affiliations:** 1 Department of Radiology, Korea University Anam Hospital, Korea University College of Medicine, 73 Goryeodae-ro, Seongbuk-gu, Seoul, 02841 Republic of Korea; 2 Department of Radiology, Korea University Ansan Hospital, Korea University College of Medicine, 123 Jeokgeum-ro, Danwon-gu, Ansan-si, Gyeonggi-do 15355 Republic of Korea; 3 Department of Radiology, Korea University Guro Hospital, Korea University College of Medicine, 148 Gurodong-ro, Guro-gu, Seoul, 08308 Republic of Korea; 4 Medical Science Research Center, Korea University Ansan Hospital, 123 Jeokgeum-ro, Danwon-gu, Ansan-si, Gyeonggi-do 15355 Republic of Korea; 5 Division of Clinical Bioinformatics, Biomedical Research Institute, Seoul National University Hospital, 101 Daehak-ro, Jongno-gu, Seoul, 03080 Republic of Korea

**Keywords:** Breast neoplasms, Magnetic resonance imaging, Neoplasm staging

## Abstract

**Background:**

Computer-aided detection (CAD) can detect breast lesions by using an enhancement threshold. Threshold means the percentage of increased signal intensity in post-contrast imaging compared to precontrast imaging. If the pixel value of the enhanced tumor increases above the set threshold, CAD provides the size of the tumor, which is calculated differently depending on the set threshold. Therefore, CAD requires the accurate setting of thresholds. We aimed to compare the diagnostic accuracy of tumor size measurement using MRI and CAD with 3 most commonly used thresholds and to identify which threshold is appropriate on CAD in breast cancer patients.

**Methods:**

A total of 130 patients with breast cancers (80 invasive cancers and 50 ductal carcinoma in situ [DCIS]) who underwent preoperative MRI with CAD and surgical treatment were included. Tumor size was manually measured on first contrast-enhanced MRI and acquired by CAD using 3 different thresholds (30, 50, and 100%) for each tumor. Tumor size measurements using MRI and CAD were compared with pathological sizes using Spearman correlation analysis. For comparison of size discrepancy between imaging and pathology, concordance was defined as estimation of size by imaging within 5 mm of the pathological size. Concordance rates were compared using Chi-square test.

**Results:**

For both invasive cancers and DCIS, correlation coefficient rho (*r*) between tumor size on imaging and pathology was highest at CAD with 30% threshold, followed by MRI, CAD with 50% threshold, and CAD with 100% threshold (all *p* <  0.05). For invasive cancers, the concordance rate of 72.5% at CAD with 30% threshold showed no difference with that of 62.5% at MRI (*p* = 0.213). For DCIS, the concordance rate of 30.0% at CAD with 30% threshold showed no difference with that of 36.0% at MRI (*p* = 0.699). Compared to MRI, higher risk of underestimation was noted when using CAD with 50% or 100% threshold for invasive cancers and when using CAD with 100% threshold for DCIS.

**Conclusion:**

For CAD analysis, 30% threshold is the most appropriate threshold whose accuracy is comparable to manual measurement on MRI for tumor size measurement. However, clinicians should be aware of the higher risk of underestimation when using CAD with 50% threshold for tumor staging in invasive cancers.

## Introduction

Preoperative evaluation of tumor size is important for appropriate surgical planning in breast cancer patients, particularly when planning in breast-conserving surgery. Negative margins can affect the risk of local recurrence [1, 2], which is 2–3 times higher when associated with a positive margin than with a negative margin [3]. Multimodal breast imaging modalities are used for the preoperative assessment of tumor size and magnetic resonance imaging (MRI) is recognized as the most accurate imaging modality [4–8]. However, MRI had a substantial risk of overestimation, which can lead to upstaging of the type of surgery [9–11]. In addition, inter- and intra-observer variations when measuring tumor size are another drawback of MRI [12].

Breast evaluation using MRI takes considerable time and experience for image processing and manual kinetic curve analysis. In addition, breast MRI has a low specificity and a high false positive rate [13]. To overcome the limitations of breast MRI and to provide an easier way of interpreting enhancement characteristics, computer-aided detection (CAD) was developed and has been widely used since 2008 [13]. It provides quantitative tumor information such as tumor size, angio-volume and kinetic curve analysis, and improves interpretation efficiency of radiologists by speeding up image processing and analysis. Moreover, it improves the specificity of MRI by determination of benign and malignant lesions using kinetic curve assessment. For accurate use of CAD, setting of an enhancement threshold is important because CAD detects breast lesions by using an enhancement threshold. Threshold means the percentage of increased signal intensity in post contrast-enhanced imaging compared to pre-contrast imaging. If a pixel value of enhancing tumors increases above a set threshold, CAD assigns a specific color to each pixel of enhancing tumors. When the tumor have presence of a color overlay on the angio-map, CAD provide the calculated size of the enhancing tumor [13]. Low threshold values can yield many false-positive pixel predictions and overestimate the tumor size, whereas high threshold values can limit the sensitivity when showing the full lesion size and underestimate the tumor size [14]. Therefore, the appropriate use of CAD requires the accurate setting of thresholds.

To balance the sensitivity and specificity and to discriminate benign from malignant breast lesions, the most appropriate threshold for CAD was suggested as 50–60% [15; 16]. Therefore, most studies using CAD use the 50% threshold [15–20]. However, in our clinical practice, we have found that the extent of enhancing tumor analyzed with CAD can be differently calculated depending on the set threshold. For instance, when using the 100% threshold on CAD, calculated tumor sizes were much smaller than those using the 30% threshold or tumors were not detected by CAD because tumors couldn’t increase above the 100% threshold. This issue has been raised by a previous research insisting that CAD can’t replace the radiologist’s assessment because it can cause negative enhancement of breast tumors [20].

Therefore, the purposes of this study were to compare tumor size measurement using MRI and CAD with the 3 most commonly used thresholds with pathological tumor size and to identify which threshold is appropriate on CAD for preoperative tumor size measurement in invasive cancers and DCIS.

## Materials and methods

### Patients

This retrospective study was performed with institutional review board approval. Between August 2008 to June 2012, we identified 160 consecutive breast cancer patients who underwent preoperative breast MRI and were processed by CAD before surgery in our institution. We excluded 39 patients (a) who underwent neoadjuvant chemotherapy before surgery (*n* = 13), (b) had multifocal or multicentric tumors that were clearly separated from each other at MRI (*n* = 10), (c) had no angio-maps because CAD did not manage large breast with matrix sizes greater than 512 × 512 × 256 (*n* = 8), or (d) had no exact tumor size on pathology report (*n* = 8). A total of 121 patients met these criteria and 9 patients had bilateral breast cancers. A total of 130 breast cancers—80 invasive cancers and 50 ductal carcinoma in situ (DCIS)—were included. The patients’ mean age was 51 years (range, 31–79 years).

We reviewed medical records to identify the presence of local recurrence after treatment. For determining molecular subtypes and adjuvant systemic chemotherapy, immunohistochemistry was done for all cancers. Local recurrence, defined as recurrence in the ipsilateral breast or chest, was determined when histological type and receptor status was concordant between primary, and recurrent cancer. The last date of data collection was February 19, 2019. The median follow-up duration was 84.0 months (range, 5.9–99.2 months).

### MRI examination

All breast MRI examinations were performed with the patients in the prone position using a 3.0-T MR imaging system (Achieva 3.0 T TX; Philips Healthcare, Best, the Netherlands) equipped with dedicated four-element SENSE-compatible breast surface coils (MRIDevices; InVivo Research, Orlando, Fla). Dynamic bilateral sagittal T1-weighted three-dimensional gradient echo images were acquired before and after contrast media injection with a total of six dynamic acquisitions. Twenty ml of 0.5 mmol/ ml Gadodiamide (Omniscan; Nycomed-Amersham, Princeton, NJ, USA) was injected intravenously followed by a 20-ml saline flush at the rate of 2.0 ml/sec. Five contrast-enhanced images were obtained at 60, 120, 180, 240, and 300 s. Axial and coronal 3D reconstruction images with maximum intensity projection were obtained (TR/TE 3.4/1.3, flip angle 10°, field of view 320 × 320 mm^2^, acquired voxel size 0.91 × 0.91 × 2.00 mm^3^, reconstructed voxel size 0.83 × 0.83 × 1.00 mm^3^).

### CAD system

**Fig. 1:**
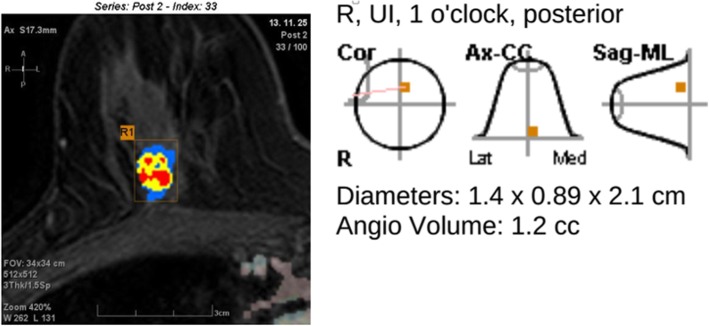
CAD angio-map showing how CAD provided the three-plane measured sizes using coronal, axial, and sagittal images

**Fig. 2:**
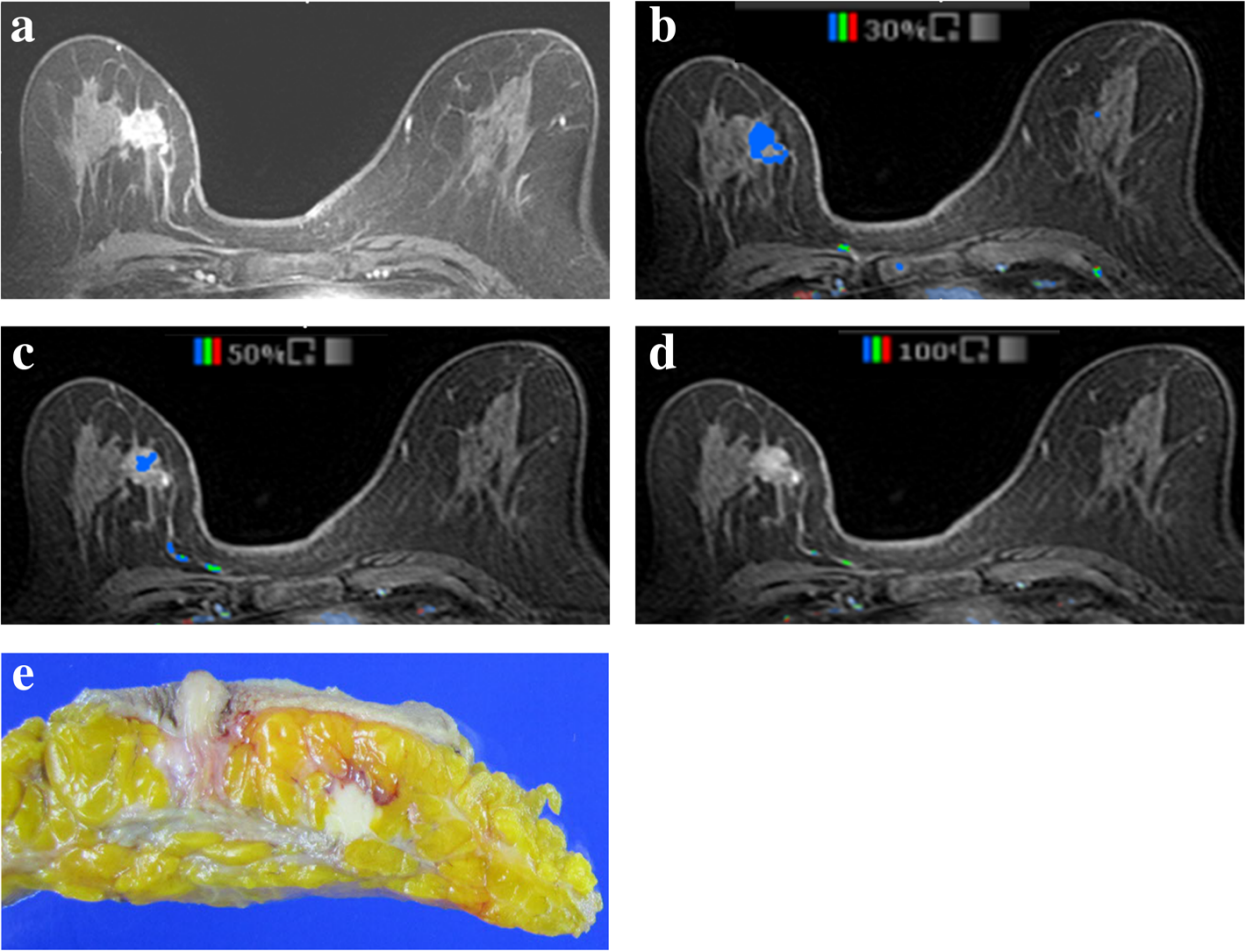
MR images of a 73-year-old-woman with invasive ductal carcinoma. **a** Axial fat-suppressed dynamic contrast-enhanced T1-weighted image depicts a 19 mm-sized irregularly shaped, irregularly marginated, heterogeneous enhancing mass at right middle-inner breast. **b** and **c** In the same image, the computer-aided detection system displayed the mass as the presence of color overlay that measured 17 mm at the 30% threshold and 9 mm at the 50% threshold. **d** The enhancing mass was not detected at the 100% threshold. **e** Actual pathologic tumor size was 22 mm on gross specimen

**Fig. 3:**
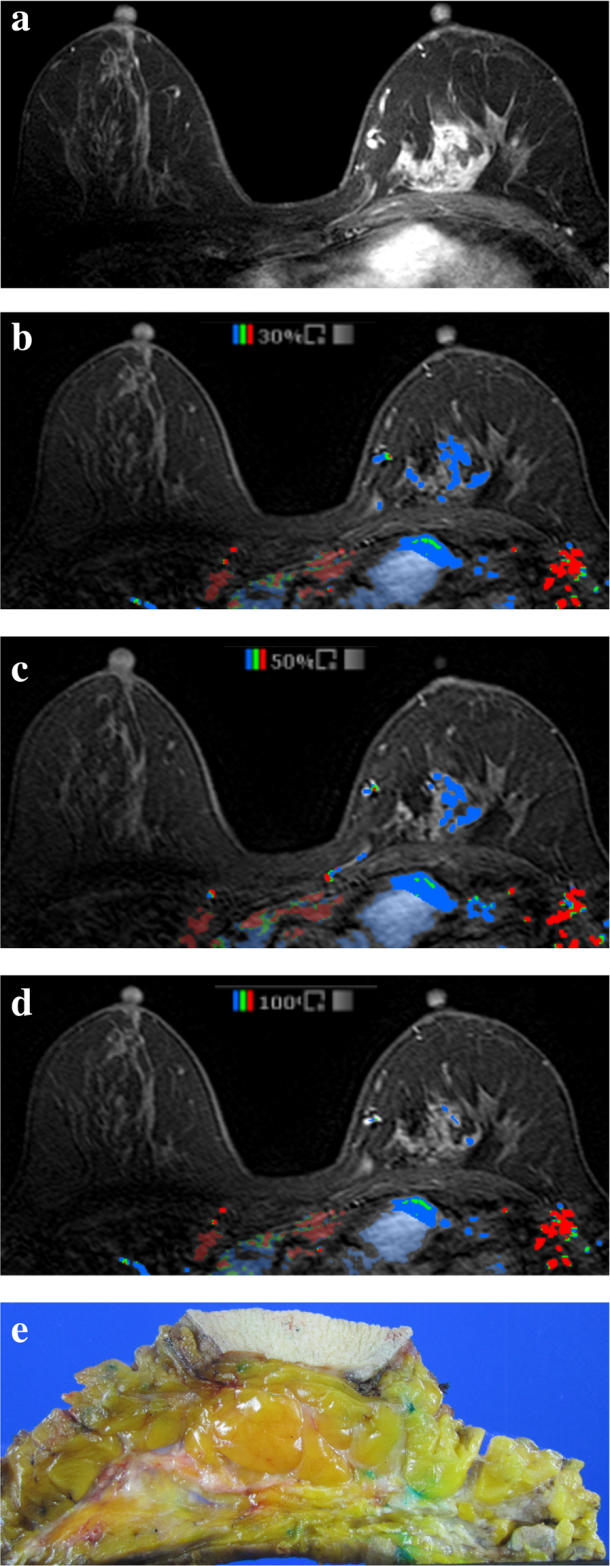
MR images of a 54-year-old-woman with ductal carcinoma in situ. **a** Axial fat-suppressed dynamic contrast-enhanced T1-weighted image shows 43 mm-sized regionally distributed non-mass enhancement in the left middle-inner breast. **b-d** In the same image obtained with a computer-aided detection system, the non-mass enhancement indicated by the presence of color overlay was measured as 34 mm at the 30% threshold, 24 mm at the 50% threshold, and 21 mm at the 100% threshold. **e** Actual pathologic tumor size was 38 mm on gross specimen

For CAD analysis, all MRI images were transferred to a commercially available CAD system (CADstream, Confirma Inc. Bellevue, WA). This workstation compared pixel intensity values on the precontrast and first contrast-enhanced series to classify enhancement. If the pixel value increased above a set enhancement threshold, CAD assigned a specific color to each pixel of tumors. If a pixel value of tumor did not increase by the established threshold, no color enhancement was made on the tumor. When the tumor above the set threshold had a presence of color overlay on the angio-map, CAD semi-automatically calculated tumor size using coronal, axial, and sagittal images and provided the three-plane measured sizes (Fig. 1). Some areas outside the breast such as blood vessels were also colored on the angio-map, but CAD was trained to accurately recognize the area of enhancement of the breast and did not include it in tumor size measurements. For CAD analysis, we applied 3 thresholds (30, 50, and 100%) which were most commonly used in clinical practice and acquired the tumor sizes calculated by CAD at each threshold (Figs. 2 and 3). Among three- plane measured sizes of tumors generated by CAD, we selected the longest tumor size.

### Imaging evaluation

On breast MRI, MRI lesion type and tumor size measurements were evaluated by 1 breast imaging radiologist (S.E.S., with 9 years’ experience) who was blinded to the clinicopathologic information. The lesion type (mass or non-mass enhancement [NME]) was described according to the Breast Imaging Reporting and Data System Atlas for MRI [21]. The three-dimensional sizes of tumors were manually measured on the first contrast-enhanced MRI using coronal, axial, and sagittal images in the same way as CAD and then, we selected the longest diameter of each tumor for comparison with tumor size by CAD.

### Pathology evaluation

Of the 130 breast cancer cases, 67 underwent breast-conserving surgery and 63 underwent a mastectomy. As soon as possible after surgery, the specimens were quickly delivered to the pathology department, sectioned, placed in 10% neutral buffered formalin, paraffin embedded, and stained with hematoxylin and eosin [22]. Along the vertical direction of the connecting line between the tumor and the nipple, sections were made every 5 mm for breast-conserving specimens and every 10 mm for mastectomy specimens. Pathological tumor size was recorded by experienced pathologists with 8–20 years’ experience. For breast-conserving specimens, the largest size was determined by measuring the stained slides directly and fitting adjacent blocks containing the tumor. For mastectomy specimens, the largest size was measured by sampling the tissue with the largest sections [23]. For invasive cancers, the longest diameter of the invasive tumor alone and the total tumor size (both invasive and in situ carcinoma components) was recorded. For DCIS, the size of the in situ carcinoma components was assessed by counting the sections that included DCIS and by measuring the maximal diameter of the lesion on the mounted sections [23].

### Statistical analysis

Spearman correlation analysis was used to calculate the linear association (correlation coefficient rho [*r*]) between tumor size measurement with MRI or CAD and pathological tumor sizes. A paired *t* test was used to compare the mean difference in tumor size measurement on imaging and pathological tumor size.

To define concordance, underestimation, and overestimation, the size discrepancy was estimated as follows. The tumor size measured with MRI or CAD subtracted from the pathological tumor size. If the size discrepancy was ≤ ± 5 mm, the measured tumor size was classified as a concordance [24]. If the measured tumor size underestimated the pathological tumor size by > 5 mm, the measured tumor size was classified as underestimation [24]. If the measured tumor size overestimated pathological tumor size by > 5 mm, the measured tumor size was classified as overestimation [24]. The chi-square test was used to compare the proportions of underestimation, concordance, and overestimation for each CAD threshold with those obtained with manual measurement on MRI as the reference standard, and was also used to compare the local reccurence rate between invasive cancers and DCIS. These analyses were performed using SPSS Statistics for Windows, version 20 (IBM Corp.).

Test of significance for correlations was additionally done for comparing the statistical difference between the two independent correlations acquired from Spearman correlation analysis. Bland–Altman limits of agreements (LOA) were also used to compare the differences between the tumor sizes on MRI or CAD and the pathological tumor sizes (reference standard) versus their mean. These two analyses were done with R version 3.0.2; *P* values < 0.05 were considered significant. This retrospective study was approved by our institutional review board, and the requirement for obtaining informed consent was waived.

## Results

### Lesions and follow-up

Pathologically, the most invasive cancers were invasive ductal carcinomas (73 of 80, 91.3%), and the other cancers (7 of 80, 8.7%) were 5 invasive lobular carcinomas, 1 invasive apocrine carcinoma, and 1 invasive cribriform carcinoma. Of the 80 invasive cancers, 72 (90.0%) cancers had absent or focal DCIS and remaining 8 (10.0%) had extensive DCIS. Of the 80 invasive cancers, 76 (95.0%) were masses and the other 4 (5.0%) were NME. Of the 50 DCIS, 25 (50.0%) were masses and 25 (50.0%) were NME.

For the 80 invasive cancers and the 50 DCIS cases, positive and negative enhancement at MRI and CAD with 3 different thresholds were summarized in Table 1. Negative enhancement meant there was no enhancing areas in the breast. For the 80 invasive cancers, 1 case showed no enhancement at the 50% threshold and 23 cases showed no enhancement at the 100% threshold. For the 50 DCIS cases, 4 cases showed no enhancement at the 50% threshold and 21cases showed no enhancement at the 100% threshold.

**Table 1:** Positive and negative enhancement at MRI and CAD in 130 breast cancers

80 Invasive cancers	Manual measurement on MRI^a^	CAD ^b^ with 30% threshold	CAD with 50% threshold	CAD with 100% threshold
Positive enhancement	80 (100.0) *	80 (100.0)	79 (98.8)	57 (71.3)
Negative enhancement	0 (0.0)	0 (0.0)	1 (1.2)	23 (28.7)
Total Number	80 (100.0)	80 (100.0)	80 (100.0)	80 (100.0)
**50 ductal carcinoma in situ**	**Manual measurement on MRI**	**CAD with 30% threshold**	**CAD with 50% threshold**	**CAD with 100% threshold**
Positive enhancement	50 (100.0)	50 (100.0)	46 (92.0)	29 (58.0)
Negative enhancement	0 (0.0)	0 (0.0)	4 (8.0)	21 (42.0)
Total Number	50 (100.0)	50 (100.0)	50 (100.0)	50 (100.0)

* Data are numbers of cancers with percentages in parentheses

^a^*MRI* magnetic resonance imaging, ^b^*CAD* computer-aided detection

Follow-up examination showed that local recurrence had occurred in 7 of the 130 cancers (5.4%). The local recurrence was more frequent for the invasive cancers (7.5%, 6 of 80) than for DCIS (2%, 1 of 50) (*p* = 0.249). The lesion type of the 7 cases of local recurrence were all masses. Among these 7 cases with local recurrence, all were MRI-concordant cases. However, 1 case was underestimated at both the 30 and 50% thresholds of CAD, and 3 cases were underestimated at the 100% threshold of CAD.

### Correlation and concordance rate in the 80 invasive cancers

For the 80 invasive cancers, the correlation coefficient was highest at CAD with 30% threshold (*r* = 0.808), followed by manual measurement on MRI (*r* = 0.778), CAD with 50% threshold (*r* = 0.729), and CAD with 100% threshold (*r* = 0.386) (all *p* < 0.001) (Table 2).

**Table 2:** Tumor sizes and correlation coefficients in 80 invasive cancers

	Pathology	Manual measurement on MRI ^a^	CAD ^b^ with 30% threshold	CAD with 50% threshold	CAD with 100% threshold
Mean tumor size (mm) (*p* value^*^)	22.47 ± 13.79	22.77 ± 12.69 (0.726)	22.35 ± 13.96 (0.887)	19.15 ± 13.75 (< 0.001)	9.91 ± 11.55 (< 0.001)
Median tumor size (mm)	20.00	20.00	18.00	15.00	6.50
Size range (mm)	5–85	6–69	5–68	0–66	0–63
Correlation coefficient (*p* value^†^)		0.778 (< 0.001)	0.808 (< 0.001)	0.729 (< 0.001)	0.386 (< 0.001)
Test of difference between two independent correlations (*p* value^††^)			(0.921)	(0.080)	(< 0.001)

* A paired t test was used to compare the mean difference in tumor size measurement with MRI or CAD and pathological tumor size

**†** Spearman correlation analysis was used to calculate the correlation coefficient between tumor size measurements with MRI

or CAD and pathological tumor size

**††** Test of difference between two independent correlations was used to test the difference between MRI and CAD with the 30% threshold or between MRI and CAD with the 50% threshold or between MRI and CAD with the 100% threshold

^a^*MRI* magnetic resonance imaging, ^b^*CAD* computer-aided detection

The concordance rate of 72.5% at CAD with 30% threshold showed no difference with that of 62.5% at manual measurement on MRI (*p* = 0.213). However, underestimation rate of 35.0% at CAD with 50% threshold and that of 71.2% at CAD with 100% threshold were higher than that of 16.2% on manual measurement on MRI (*p* = 0.036, and *p* < 0.001, respectively) (Table 3).

**Table 3:** Concordance rates of MRI and CAD in 80 invasive cancers

	Manual measurement on MRI ^a^	CAD ^b^ with 30% threshold	CAD with 50% threshold	CAD with 100% threshold
Underestimation	13 (16.2)	12 (15.0)	28 (35.0)	57 (71.2)
Concordance	50 (62.5)	58 (72.5)	43 (53.8)	23 (28.7)
Overestimation	17 (21.3)	10 (12.5)	9 (11.2)	0 (0.0)
Total Number	80 (100.0)	80 (100.0)	80 (100.0)	80 (100.0)
*p* value**†**		0.213	0.036	< 0.001

* Data are numbers of cancers with percentages in parentheses

**†** The chi-square test was used to compare the outcomes of CAD with that those of MRI as a reference standard

^a^*MRI* magnetic resonance imaging, ^b^*CAD* computer-aided detection

**Fig. 4:**
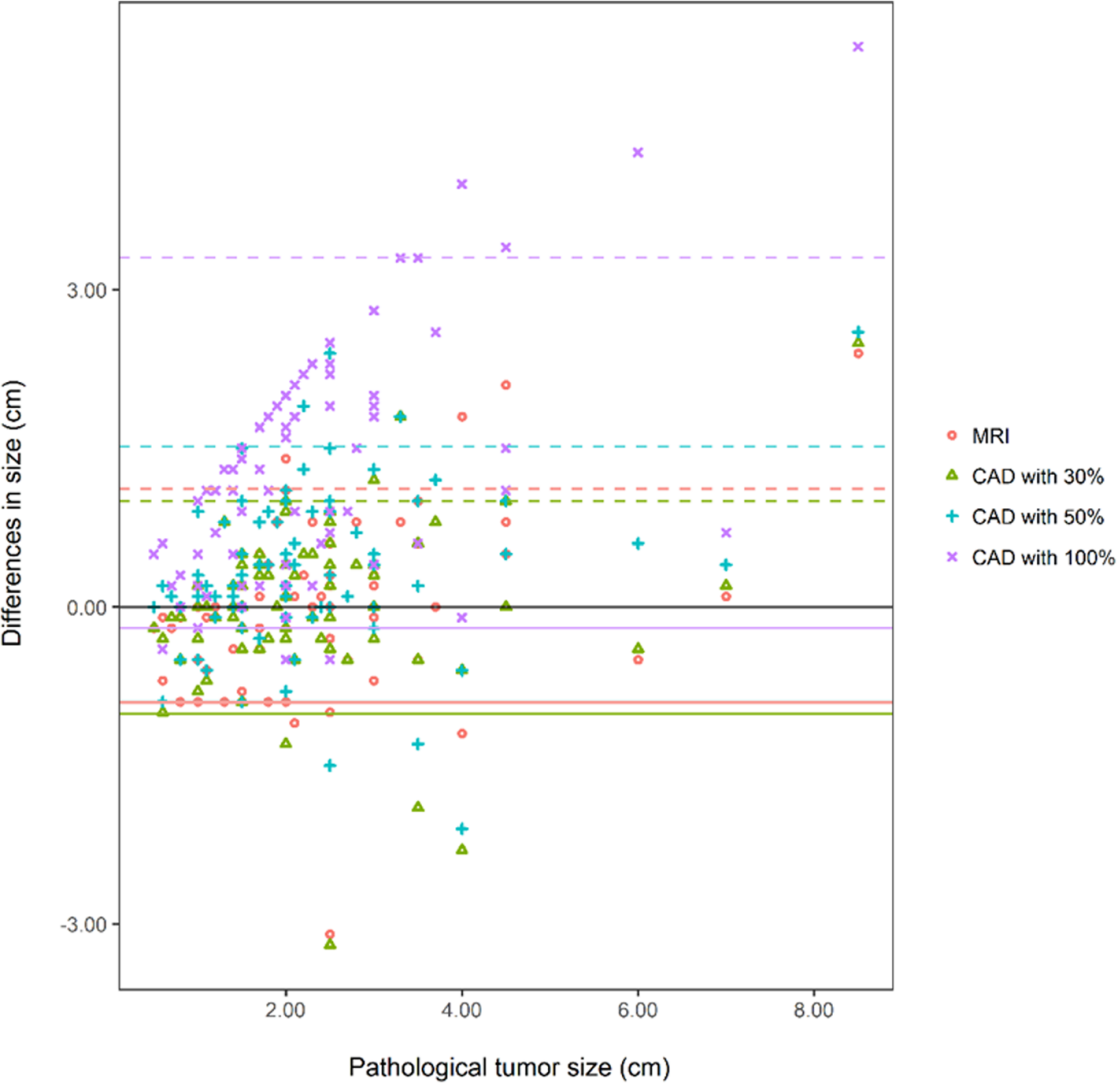
The Bland-Altman plot for comparison of the differences between the tumor sizes on MRI or CAD and the pathological tumor sizes in 80 invasive cancers. Times indicates MRI size and pathological tumor size. Triangle indicates CAD with 30% threshold. Cross indicates CAD with 50% threshold. X mark indicates CAD with 100% threshold. This plot shows that that the limits of agreements of CAD with 30% threshold are narrower compared with those of the other imaging modalities

The Bland–Altman plot showed that the LOA of CAD with 30% threshold were narrower compared with those of the other imaging modalities (Fig. 4).

### Correlation and concordance rate in the 50 DCIS cases

For the 50 DCIS cases, the correlation coefficient was highest at CAD with 30% threshold (*r* = 0.659), followed by manual measurement on MRI (*r* = 0.620), CAD with 50% threshold (*r* = 0.600), and CAD with 100% threshold (*r* = 0.370) (all *p* < 0.05) (Table 4).

**Table 4:** Tumor sizes and correlation coefficients in 50 ductal carcinoma in situ

	Pathology	Manual measurement on MRI ^a^	CAD ^b^ with 30% threshold	CAD with 50% threshold	CAD with 100% threshold
Mean tumor size (mm) (*p* value^*^)	24.96 ± 17.97	25.80 ± 15.50 (0.678)	27.06 ± 17.00 (0.297)	21.54 ± 17.45 (0.118)	8.28 ± 12.29 (< 0.001)
Median tumor size (mm)	21.00	23.50	23.00	17.00	4.00
Size range (mm)	4–90	4–78	6–84	0–81	0–60
Correlation coefficient (*p* value^†^)		0.620 (< 0.001)	0.659 (< 0.001)	0.600 (< 0.001)	0.370 (0.002)
Test of difference between two independent correlations (*p* value^††^)			(0.810)	(0.890)	(0.120)

* A paired t test was used to compare the mean difference in tumor size measurement with MRI or CAD and pathological tumor size

**†** Spearman correlation analysis was used to calculate the correlation coefficient between tumor size measurements with MRI

or CAD and pathological tumor size

**††** Test of difference between two independent correlations was used to test the difference between MRI and CAD with the 30% threshold or between MRI and CAD with the 50% threshold or between MRI and CAD with the 100% threshold

^a^*MRI* magnetic resonance imaging, ^b^*CAD* computer-aided detection

The concordance rates of 30.0% at CAD with 30% threshold and 34.0% at CAD with 50% threshold showed no difference with that of 36.0% at manual measurement on MRI (*p* = 0.699 and *p* = 0.744, respectively). However, underestimation rates of 76.0% at CAD with 100% threshold was higher than that of 32.0% on manual measurement on MRI (*p* < 0.001) (Table 5).

**Table 5:** Concordance rates of MRI and CAD in 50 ductal carcinoma in situ

	Manual measurement on MRI ^a^	CAD ^b^ with 30% threshold	CAD with 50% threshold	CAD with 100% threshold
Underestimation	16 (32.0) *	15 (30.0)	20 (40.0)	38 (76.0)
Concordance	18 (36.0)	15 (30.0)	17 (34.0)	9 (18.0)
Overestimation	16 (32.0)	20 (40.0)	13 (26.0)	3 (6.0)
Total number	50 (100.0)	50 (100.0)	50 (100.0)	50 (100.0)
*p* value**†**		0.699	0.744	< 0.001

* Data are numbers of cancers with percentages in parentheses

**†** The chi-square test was used to compare the outcomes of CAD with that those of MRI as a reference standard

^a^*MRI* magnetic resonance imaging, ^b^*CAD* computer-aided detection

**Fig. 5:**
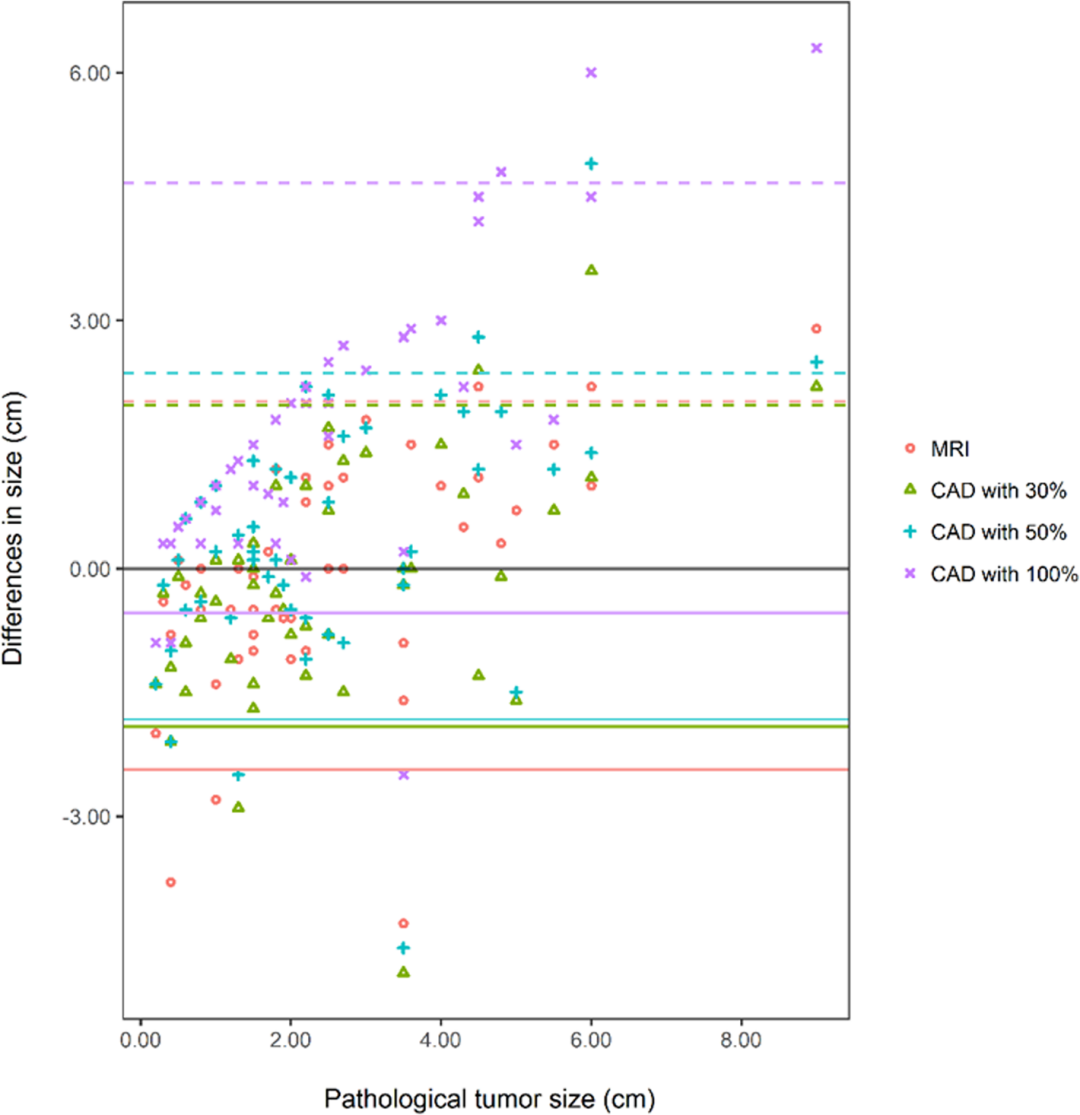
The Bland-Altman plot for comparison of the differences between the tumor sizes on MRI or CAD and the pathological tumor sizes in 50 ductal carcinoma in situ. Times indicates MRI size and pathological tumor size. Triangle indicates CAD with 30% threshold. Cross indicates CAD with 50% threshold. X mark indicates CAD with 100% threshold. This plot shows that that the limits of agreements of CAD with 30% threshold are narrower compared with those of the other imaging modalities

The Bland–Altman plot showed that the LOA of CAD with 30% threshold were narrower compared with those of the other imaging modalities (Fig. 5).

## Discussion

Breast MRI is considered to be the most accurate imaging modality for preoperative tumor size measurement in breast cancer patients. However, it also has disadvantages such as over- or underestimation of the tumor size [5, 7, 9–11, 25, 26] and inter- and intra-observer variations [12]. To overcome these shortcomings, CAD is used as an alternative for quantifying the tumor size. However, only a few studies have investigated tumor size measurement using CAD for invasive cancers [8, 27, 28]. Whereas two studies using the 50% threshold reported that CAD was feasible for preoperative staging [8] and for predicting the residual tumor size after neoadjuvant chemotherapy [28], another study using the 100% threshold concluded that CAD is less accurate [27]. These inconsistent results may reflect the use of different threshold settings, which are the most important parameter when using CAD. Previous studies have reported that the threshold should not be set at < 50% because of the risk of high false-positive rates and should not be set at > 60% because of the limited ability to identify the full lesion size [14, 15]. Hence, the commonly used threshold in clinical practice is 50%, although the user can select the threshold from the range of 30 to 200% [8, 15–20, 27, 28]. To determine the appropriate thresholds for the use of CAD in preoperative tumor size measurement, further studies comparing the tumor size measurements using different thresholds for each type of tumor are required. To our knowledge, the present study is the first to assess the accuracy of tumor size assessment with MRI and CAD using 3 thresholds for both invasive cancers and DCIS.

For invasive cancer, CAD with 30% threshold showed the best performance outcomes for correlation coefficient, concordance rate, and underestimation rate. When using CAD with 50% or 100% threshold, underestimation rates were significantly higher than that of manual measurement on MRI. However, the underestimation rate of 15.0% at CAD with 30% threshold was similar to that of 16.2% at manual measurement on MRI. Taken together, these data suggest that CAD with 30% threshold is the most precise tool and is comparable to MRI without the risk of underestimation. In our study, the observation that the 100% threshold had a significantly higher underestimation rate was expected from previous studies that have reported limited sensitivity of the 100% threshold at CAD [14, 15]. We also note that the 50% threshold which has been most widely used in clinical practice had a significantly higher underestimation rate of 35.0%, which was more than twice as high as the 16.2% for MRI.

MRI is increasingly used for the preoperative staging in DCIS [7, 25, 29]. Our correlation coefficients for MRI or for the 30% or 50% threshold at CAD were all within the range of 0.409 to 0.786, as reported from previous studies [7, 29]. However, both the correlation coefficients and concordance rates were considerably lower for DCIS than for invasive cancers, and these low rates may reflect the inherent characteristics of DCIS [9, 25, 30–32]. In terms of morphologic feature, NME rather than masses is the dominant feature of DCIS [25, 30–32]. In a previous study of the factors influencing discordance of tumor size measurements [9], only NME significantly predicted discordance. Moreover, for kinetic feature, NME had less suspicious enhancement kinetics than mass lesions [31, 32]. Therefore, we conclude that, for DCIS, tumor size measurement with CAD using the 30% or 50% threshold should be able to achieve equivalent accuracy in predictions as that using MRI; however, the results were inferior to those for invasive cancers probably because of the inherent characteristics of DCIS.

The issue of overestimation with the use of MRI to measure tumor size has been consistently raised. Although some studies have reported that MRI overestimates the pathological tumor size [5, 7, 33], other studies have reported that MRI underestimates the pathological size [25, 26]. In the MRI analysis of our study, overestimation was more frequent than underestimation in the invasive cancers, and both overestimation and underestimation took place with similar probabilities in the DCIS cases. Interestingly, the overestimation rate decreased and concordance rate increased for invasive cancers when analyzed using the 30% threshold at CAD. This may reflect the interconnection between enhancement of high-risk lesions and proliferative disease associated with invasive tumors, which has also been suggested as a reason for overestimation by MRI, are not as well recognized by CAD because they do not enhance above a set threshold of CAD [34]. Accordingly, the application of CAD with 30% threshold may help to decrease the high overestimation rate of invasive breast cancers when analyzed using MRI.

Underestimation is also great importance because it may lead to inadequate treatment, which can influence the risk of local recurrence. In our study, we observed negative enhancement of tumors at CAD with 50% or 100% thresholds. Not only negative enhancement but also underestimation of tumor sizes by CAD can limit the use of CAD in the preoperative tumor staging because it can affect the local recurrence. In the present study, 7 cases of local recurrence were found on follow-up; of these 7 cases, 3 cases had been underestimated at the 100% threshold of CAD and 1 had been underestimated at both the 30 and 50% thresholds. Therefore, when using CAD in preoperative tumor size measurement, it is essential to be aware of the higher risk of underestimation which may influence the local recurrence.

Although our preliminary results are promising, our study has several limitations. First, this was a retrospective review of records from a single institution. Second, the tumor extent was measured manually on MRI by only 1 experienced breast radiologist, and the lack of investigation of inter- and intraobserver agreement variations limits the power of this study. In this study, we did focus on availability of CAD with different thresholds. Third, tumor size measurements were taken by only CADstream in our institution and the possibility of different results when using other CAD program cannot be excluded. However, both CADstream and DynaCAD (Invivo corp, Orlando, FL), which are the two most widely used CAD programs among all currently available systems, display the suspicious tissues based on contrast enhancement level above a set threshold in a similar way [13, 35–37]. Fourth, we did not investigate the yield of false-positive pixel predictions when using the 30% threshold, although it is known that low threshold values can produce many false-positive pixel predictions of breast lesions. Lastly, we used three representative thresholds such as 30, 50 and 100%, which are commonly used in clinical practice. However, the possibility of presence of other appropriate thresholds less than 50% cannot be excluded.

## Conclusion

In conclusion, CAD is as accurate as manual measurement on MRI for preoperative tumor size estimation in breast cancer patients. Among the 3 most commonly used thresholds, 30% seems to be the most appropriate threshold and produced results similar to those of manual measurement on MRI for both invasive cancers and DCIS. However, we note a higher risk of underestimation for CAD with the 50 and 100% thresholds for invasive cancers and with the 100% threshold for DCIS. Our data analysis may help contribute to the consensus about the proper use of MRI and CAD for preoperative staging work-up in breast cancer patients.

## Data Availability

Please contact authors for data requests.

## Abbreviations

CAD: Computer-aided detection
MRI: Magnetic resonance imaging
DCIS: Ductal carcinoma in situ
NME: Non-mass enhancement
